# Supplementary figures and images for: Toll-like receptor 7-adapter complex modulates interferon-α production in HIV-stimulated plasmacytoid dendritic cells
Source: PLoS One. 2019 Dec 12;14(12):e0225806. doi: 10.1371/journal.pone.0225806 (PMC6907767; doi:10.1371/journal.pone.0225806)

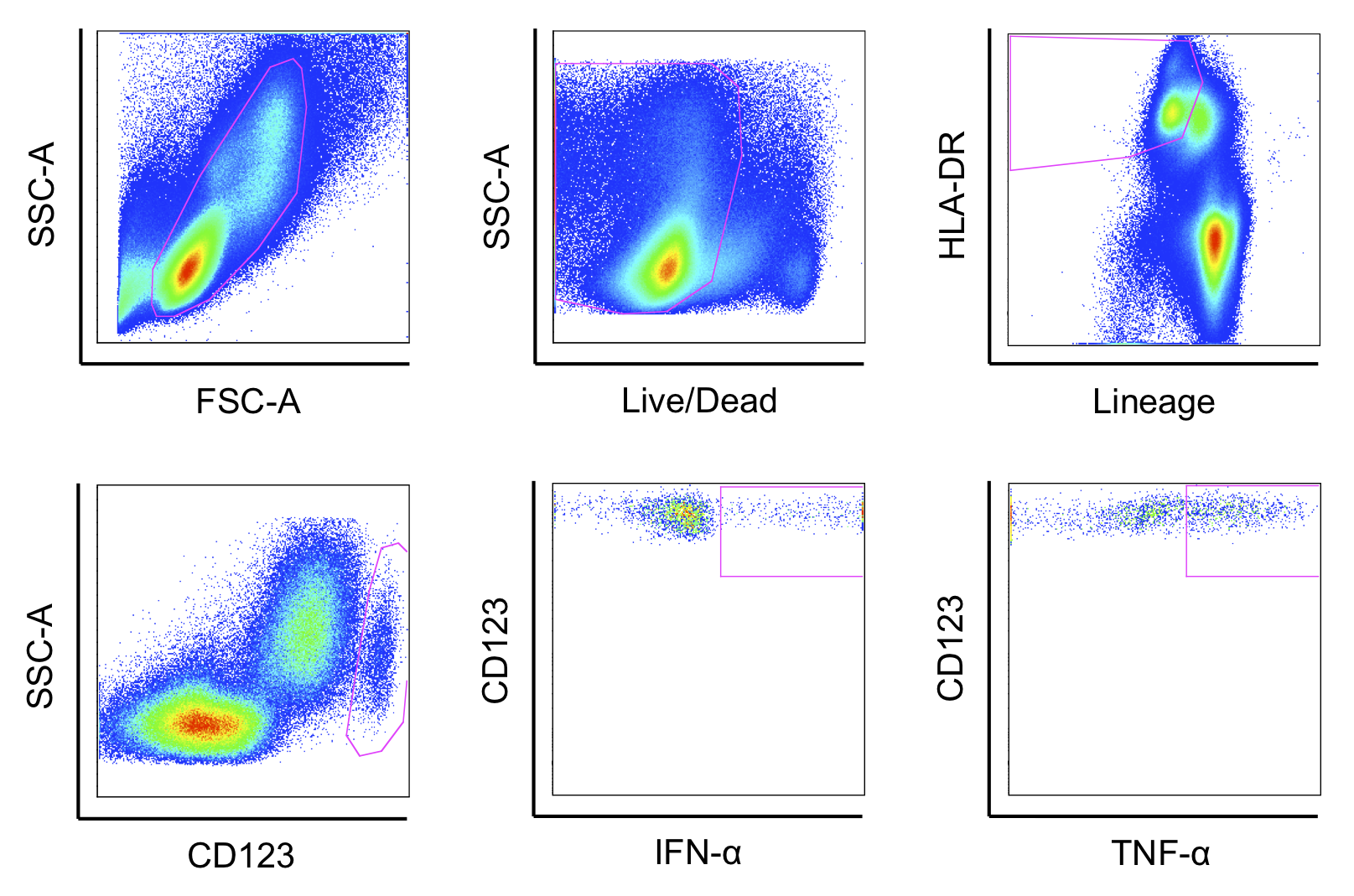

Supplement: S1 Fig — Total PBMCs were gated by Forward Scatter Area (FSC-A) vs. Side Scatter Area (SSC-A). Dead cells were excluded by Live/Dead fixable violet dead cell stain vs. SSC-A. PDC populations are lineage-, HLA-DR+, and CD123+. The production of IFN-α and TNF-α in PDCs was measured following an 8 hour stimulation. (TIF) [file pone.0225806.s001.tif]

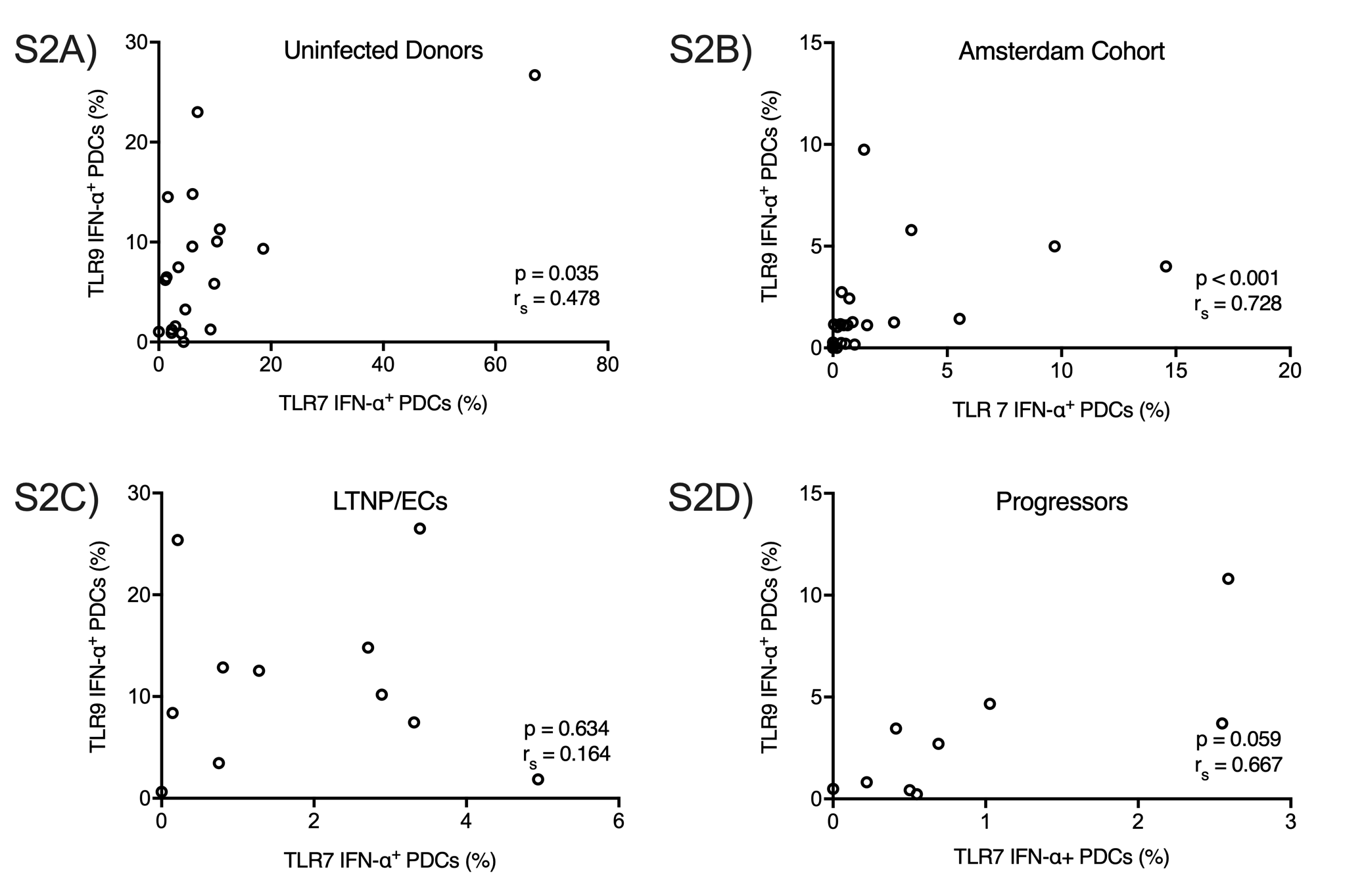

Supplement: S2 Fig — PDC TLR7 receptors were stimulated by HIV-1ADA, and PDC TLR9 receptors were stimulated by CpG A. Cytokine production was inhibited after 4 hours. An anti-IFN-α antibody was used to measure intracellular IFN-α production by flow cytometry. P and rs values were calculated by the Spearman rank correlation. (TIF) [file pone.0225806.s002.tif]

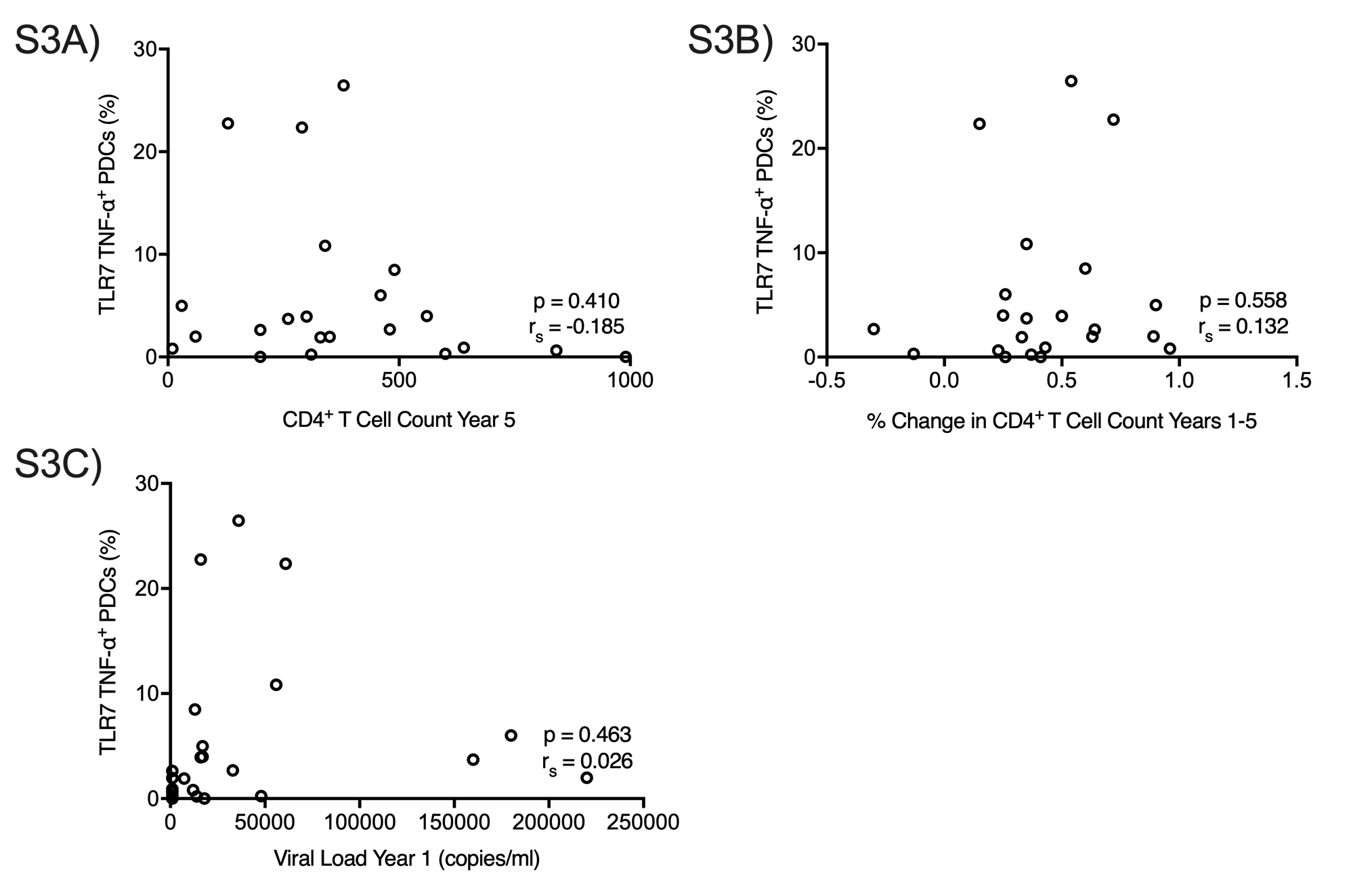

Supplement: S3 Fig — PDC TLR7 receptors were stimulated by HIV-1ADA, and cytokine production was inhibited after 4 hours. An anti-TNF-α antibody was used to measure intracellular TNF-α production by flow cytometry. TNF-α production by PDCs was correlated with either the number of CD4+ T cells 5 years post-infection, the percent change in the number of CD4+ cells from 1 to 5 years post infection, or the viral load in year 1. P and rs values were calculated by the Spearman rank correlation. (TIF) [file pone.0225806.s003.tif]
