# Supplementary material for: Toll-like receptor 7-adapter complex modulates interferon-α production in HIV-stimulated plasmacytoid dendritic cells
Source: PLoS One. 2019 Dec 12;14(12):e0225806. doi: 10.1371/journal.pone.0225806 (PMC6907767; doi:10.1371/journal.pone.0225806)
Supplement: Attachment — Submitted filename: Response to Reviewers.docx [file pone.0225806.s004.docx]

Response to Reviewers

1. Table 1: Is the plasma viral load for the progressor subjects below 50 copies/ml?

We have now clarified that the progressor participants had a history of uncontrolled viremia (to distinguish them from LTNP/EC) but were stably suppressed to <50 copies/ml at the time of measurement in the Methods and legend to Table 1.

2.	For clarity, the authors should name the panels in the figures and refer to them when describing the results.

The panels in figures with multiple graphs have been labeled and additional details were added to the results section where figures are referenced.

3.	Figure legends should include more details about the experiments/results

The figure legends were revised to include a result in the title and methods in the descriptions.

4.	Why did the authors use inactivated HIV particles to stimulate pDCs?

2-aldithriol-inactivated (AT-2) HIV particles have been compared to infectious particles in one study (Sabado et al., Plos One, 2019). They found that inactivated particles are slightly better for antigen presentation. Although the mechanism for this difference is unclear, we primarily used them because we with to avoid a perception of diminished pDC function possibly caused by HIV infection and their use is widespread in this field permitting direct comparisons with our results.

5.	Did the authors try to stimulate pDC with different amounts of inactivated HIV particles or RNA41 to avoid saturation of CD4 binding or in the expression pathway or INF-a?

The amount of AT-2 inactivated HIV-1 particles (HIV-1_ADA_) was titrated in preliminary work (Tilton et al., ref 15). Those titrations indicated that amounts higher than 250 ng of AT-2 inactivated HIV-1 particles did not result in greater amounts of INF-a production. Saturation would be expected to cause less of an increase in IFN-a production for each increase in particle dose, lessening inter-patient variation. However, these variations occurred despite these high doses. Use of a high amount in the present paper permits us to explore the potential mechanism regarding these variations.
